# P-1856. Desirability of Outcome Ranking Analysis Framework for Patients on Outpatient Parenteral Antimicrobial Therapy: A Proof-of-Concept Study in Immunocompromised Patients Receiving Daptomycin or Vancomycin

**DOI:** 10.1093/ofid/ofaf695.2025

**Published:** 2026-01-11

**Authors:** Elaine Kim, Madison Ponder, Luther A Bartelt, Anne Friedland, Asher J Schranz, David van Duin, Brent W Footer

**Affiliations:** MD Anderson , Houston, TX; University of North Carolina at Chapel Hill, Chapel Hill, NC; University of North Carolina School of Medicine, Chapel Hill, NC; UNC School of Medicine, Chapel Hill, NC; University of North Carolina, Chapel Hill, NC; University of North Carolina at Chapel Hill, Chapel Hill, NC; University of North Carolina Medical Center, Chapel Hill, North Carolina

## Abstract

**Background:**

Desirability of Outcome Ranking (DOOR) analysis provides a global assessment of patient outcomes by accounting for both benefits and harms in a single outcome measure. Currently, there is no standardized DOOR framework for patients on outpatient parenteral antimicrobial therapy (OPAT). The goal of this study was to propose an OPAT DOOR framework and apply it to a cohort of patients receiving either daptomycin (DAP) or vancomycin (VAN).

**Table 1.**
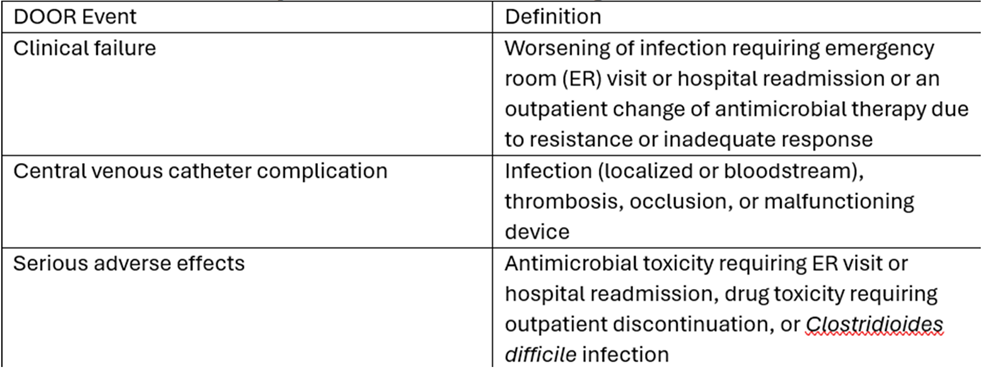
Desirability of Outcome Ranking Events and Definitions

**Table 2.**
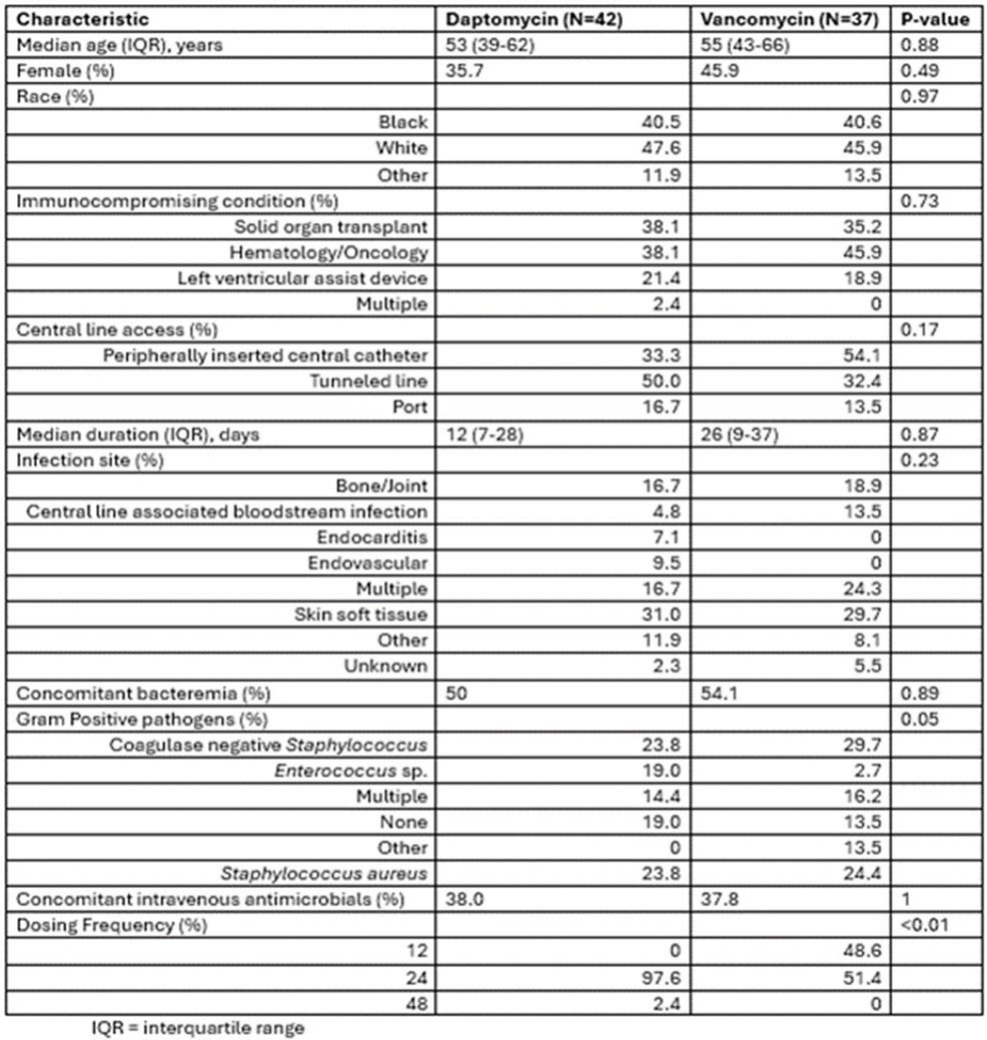
Baseline Characteristics

**Methods:**

This study included adult, immunocompromised patients from a single-center treated with either DAP or VAN via home-based OPAT between 01/01/2023-04/01/2025. Patients may have received other concomitant intravenous antimicrobials. We proposed a DOOR outcome and applied it in a comparison of DAP versus VAN.

Three DOOR events were proposed and defined as specific patient events (Table 1). Events were captured from the date of discharge through completion of intravenous antimicrobials. The DOOR outcome was defined as a ranking of the number of events experienced by a patient. Rank 1 is the most desirable outcome and included patients who were alive and did not experience an event. Ranks 2 through 4 included patients alive but who had 1, 2, or 3 events. Rank 5 was least desirable outcome and included patients who died.

**Figure 1.**
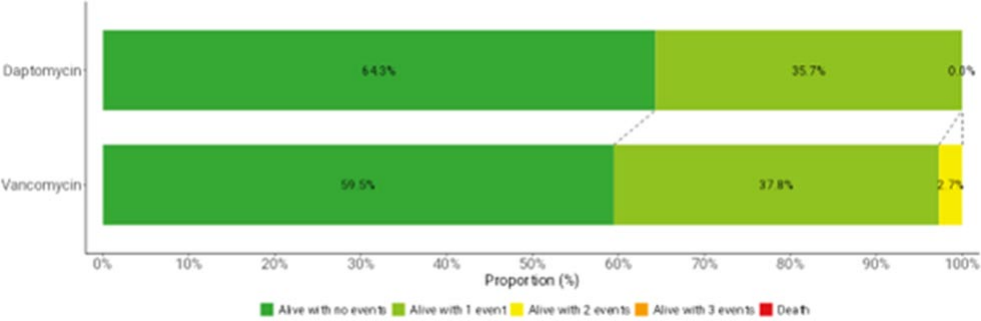
Desirability of outcome ranking (DOOR) distribution by treatment groups

**Figure 2.**
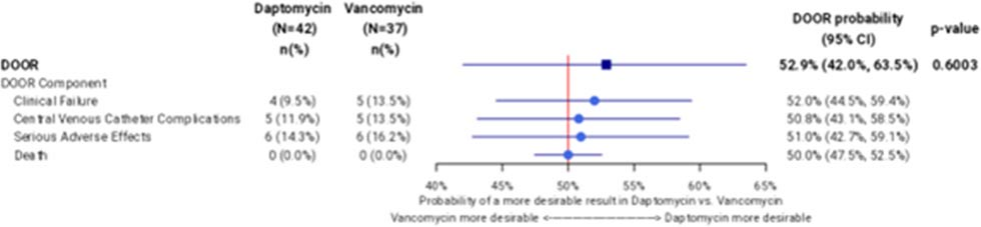
Forest plot of the probabilities for each individual desirability of outcome ranking (DOOR) events CI, confidence interval

**Results:**

A total of 79 patients were included, 42 DAP and 37 VAN. Baseline characteristics were overall similar between groups except for the specific Gram-positive pathogen (Table 2). Concomitant intravenous antibiotics were beta-lactams in all but one patient.

The DOOR distribution was similar between DAP and VAN with probability of having a more desirable outcome with DAP, compared with VAN, of 52.9% (95% confidence interval [CI], 42.0%–63.5%), indicating no significant difference (Figure 1). Additionally, no significant differences were observed between the DAP and VAN groups for any of the component undesirable events contributing to the DOOR rank (Figure 2).

**Conclusion:**

Adoption of a standardized OPAT DOOR analysis may help to better characterize potential risks when comparing therapies and allow for comparison of results across different studies. In a test case of the novel DOOR outcome, no difference was observed between patients treated with vancomycin versus daptomycin.

**Disclosures:**

Luther A. Bartelt, MD, NIH: Grant/Research Support Anne Friedland, MD, Paratek Pharmaceuticals: Grant/Research Support Asher J. Schranz, MD, MPH, Uptodate: payment for authorship David van Duin, MD, PhD, British Society for Antimicrobial Chemotherapy: Editor stipend|Merck: Advisor/Consultant|Merck: Grant/Research Support|Pfizer: Advisor/Consultant|Roche: Advisor/Consultant|Shionogi: Advisor/Consultant

